# Prospective detection of mutations in cerebrospinal fluid, pleural effusion, and ascites of advanced cancer patients to guide treatment decisions

**DOI:** 10.1002/1878-0261.12574

**Published:** 2019-10-11

**Authors:** Sergio Villatoro, Clara Mayo‐de‐las‐Casas, Núria Jordana‐Ariza, Santiago Viteri‐Ramírez, Mónica Garzón‐Ibañez, Irene Moya‐Horno, Beatriz García‐Peláez, María González‐Cao, Umberto Malapelle, Ariadna Balada‐Bel, Alejandro Martínez‐Bueno, Raquel Campos, Noemí Reguart, Margarita Majem, Remei Blanco, Ana Blasco, María J. Catalán, Xavier González, Giancarlo Troncone, Niki Karachaliou, Rafael Rosell, Miguel A. Molina‐Vila

**Affiliations:** ^1^ Laboratory of Oncology Pangaea Oncology Quirón Dexeus University Hospital Barcelona Spain; ^2^ Dr Rosell Oncology Institute Quirón Dexeus University Hospital Barcelona Spain; ^3^ Dr Rosell Oncology Institute General Hospital of Catalonia Sant Cugat del Vallés Spain; ^4^ Department of Public Health University of Naples Federico II Italy; ^5^ Department of Medical Oncology Hospital Clínic Barcelona Spain; ^6^ Medical Oncology Service Hospital de Sant Pau Barcelona Spain; ^7^ Medical Oncology Service Hospital de Terrassa Spain; ^8^ Medical Oncology Department Hospital General Universitario de Valencia Spain; ^9^ Dr Rosell Oncology Institute University Hospital Sagrat Cor Barcelona Spain; ^10^ Cancer Biology and Precision Medicine Program Catalan Institute of Oncology Germans Trias i Pujol Health Sciences Institute and Hospital Badalona Spain

**Keywords:** ascites, cell‐free DNA, cerebrospinal fluid, liquid biopsy, pleural effusion, solid tumors, somatic mutations

## Abstract

Many advanced cases of cancer show central nervous system, pleural, or peritoneal involvement. In this study, we prospectively analyzed if cerebrospinal fluid (CSF), pleural effusion (PE), and/or ascites (ASC) can be used to detect driver mutations and guide treatment decisions. We collected 42 CSF, PE, and ASC samples from advanced non‐small‐cell lung cancer and melanoma patients. Cell‐free DNA (cfDNA) was purified and driver mutations analyzed and quantified by PNA‐Q‐PCR or next‐generation sequencing. All 42 fluid samples were evaluable; clinically relevant mutations were detected in 41 (97.6%). Twenty‐three fluids had paired blood samples, 22 were mutation positive in fluid but only 14 in blood, and the abundance of the mutant alleles was significantly higher in fluids. Of the 34 fluids obtained at progression to different therapies, *EGFR* resistance mutations were detected in nine and *ALK* acquired mutations in two. The results of testing of CSF, PE, and ASC were used to guide treatment decisions, such as initiation of osimertinib treatment or selection of specific ALK tyrosine–kinase inhibitors. In conclusion, fluids close to metastatic sites are superior to blood for the detection of relevant mutations and can offer valuable clinical information, particularly in patients progressing to targeted therapies.

## Graphical abstract

Many advanced cases of cancer show central nervous system, pleural, or peritoneal involvement. Here, we present the results of prospective genetic testing of 42 cerebrospinal fluids, pleural effusions, and ascites, and we show that these fluids, which are closer to metastatic sites, are superior to plasma (P) for the detection of clinically relevant mutations.

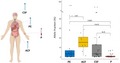

**Abbreviations**
cfDNA: cell‐free DNA
CNS: central nervous system
CSF: cerebrospinal fluid
NGS: next‐generation sequencing
NSCLC: non‐small‐cell lung cancer
PE: pleural effusion
TKI: tyrosine–kinase inhibitor

## 1 Introduction

With the advent of precision medicine, mutation testing in tumor biopsies from advanced patients has become a routine practice in several malignancies, including non‐small‐cell lung cancer (NSCLC), metastatic melanoma, or colorectal cancer. In addition, genetic analysis of the so‐called ‘liquid biopsies’, particularly blood samples, is quickly gaining acceptance in the clinical setting (Bedin *et al.*, 2017; de Wit *et al.*, 2019 Goldman *et al.*, 2018; Mayo‐de‐Las‐Casas *et al.*, 2018; Mayo‐de‐Las‐Casas *et al.*, 2017; Rolfo *et al.*, 2018). Although liquid biopsies are unlikely to replace ‘real’ biopsies, they provide valuable information that cannot be obtained in any other way. In patients where biopsies are impossible or do not yield enough tissue, biological fluids are the only samples available for genetic testing. Moreover, in patients with multiple metastases, it is not feasible to obtain biopsies of every site but genetic materials coming from all of them can be found in blood. Finally, in contrast to ‘real biopsies’, liquid biopsies can be periodically obtained with highly limited risk of complications through the procedure. This repeated testing can be used to monitor the course of the disease and facilitate early evaluation of response, detection of relapse, and characterization of resistance mechanisms to targeted therapies (Tsui *et al.*, 2018; Ulz *et al.*, 2017; Yi *et al.*, 2017). This last aspect is particularly relevant, since drugs active against some of the resistance mechanisms are approved or in late‐stage clinical trials. Examples include osimertinib, which targets the p.T790M mutation in *EGFR* emerging in NSCLC patients treated with EGFR tyrosine kinase inhibitors (TKIs); or second‐ and third‐generation ALK TKIs, such as brigatinib or lorlatinib, that show efficacy in patients harboring resistance mutations in *ALK *(Zhang *et al.*, 2016).

Circulating free DNA (cfDNA) purified from serum or plasma is the most widely used type of liquid biopsy. Since it contains a fraction of tumor DNA (circulating tumor DNA) mixed with DNA from normal cells, it is employed to identify mutations, amplifications, gene fusions, and other somatic alterations that modify the DNA sequence (Gonzalez‐Cao *et al.*, 2015; Mayo‐de‐Las‐Casas *et al.*, 2017; Szallasi, 2017). However, the concept of ‘liquid biopsy’ is not restricted to blood‐derived samples and includes other body fluids such as cerebrospinal fluid (CSF), pleural effusion (PE), and ascites (ASC). In all these cases, cfDNA can be isolated from the supernatants obtained after centrifugation of the fluid and submitted to genetic analyses. A number of studies have evaluated whether cfDNA purified from CSF, PE, and ASC can be reliably used to determine mutations and other alterations in cancer patients (Table S1) and have concluded that these fluids are suitable for molecular profiling (Li *et al.*, 2018; Xu *et al.*, 2018). However, these studies have generally been performed in a retrospective manner using fluid samples with paired biopsies and, to the best of our knowledge, a recently published case report is the only example of ‘nonblood’ liquid biopsies actually being used to orient clinical decisions (Melms *et al.*, 2018). Therefore, the performance and utility in the clinical setting of prospective molecular profiling using cfDNA from CSF, PE, and ASC remain to be established.

In this article, we describe the results of the implementation in the routine practice of mutation analysis in CSF, PE, and ASC of advanced cancer patients with central nervous system (CNS), pleural, and peritoneal involvement, respectively. A total of 42 ‘nonblood’ liquid biopsies, 34 of them at progression to therapies, were systematically collected in several institutions; cfDNA was purified and the subsequent molecular testing used to guide treatment decisions. Our work demonstrates that prospective genetic analysis of cfDNA in CSF, PE, and ASC offers valuable clinical information and that these fluids are superior to blood for mutation testing in patients with certain metastatic sites.

## 2 Materials and methods

### 2.1 Patients

Forty‐two fluids collected during a period of 2 years in eight hospitals from 23 advanced NSCLC and two metastatic melanoma patients were prospectively tested for genetic alterations in a central laboratory located in the Quirón Dexeus Hospital (Table 1). The study was carried out in accordance with the principles of the Declaration of Helsinki under an approved protocol of the institutional review board of Quirón Hospitals. Written informed consent was obtained from all patients and documented and samples were deidentified for patient confidentiality.

**Table 1:** Characteristics of the patients included in the study.

Characteristics	All patients *N* = 25
Gender	
Male	8
Female	17
Primary tumor	
Advanced NSCLC	23
Advanced melanoma	2
Type of mutation	
*EGFR*	19
*KRAS*	1
*BRAF*	3
*ALK*	2
Patients with samples	
Only at presentation	1
Only at progression	19
More than one sample	5
Presentation and follow‐up	1
Follow‐up and progression	4

### 2.2 Cell lines

NCI‐H1975, NCI‐H23, DLD1, HT29, and NCI‐H460 human tumor cell lines were purchased from the American Type Culture Collection (Rockville, MD, USA) and grown under standard conditions; PC9 was obtained from Hofmann‐La Hofmann Roche Ltd with the authorization of M. Ono. Cell lines were validated by genotyping them for *EGFR, KRAS, PIK3CA, BRAF,* and *TP53* and used as positive or negative controls (Table S3). DNA from the cell lines was purified using the DNA Easy^®^ extraction kit (Qiagen, Hilden, Germany), according to the manufacturer’s instructions, and used as a control material.

### 2.3 cfDNA isolation from fluid and plasma samples

Fluid samples (3–500 mL) were collected in sterile containers and plasma samples (10 mL) in Vacutainer tubes (BD, Plymouth, UK). After a first centrifugation step (509 ***g***, 10 min), the supernatants were transferred to a new tube and immediately submitted to a second centrifugation followed by cfDNA purification. The sediments of the first and second centrifugation, if present, were extended on slides, stained by hematoxylin and examined under a microscope. Purification of cfDNA from supernatants (1.2 mL) was performed with the QIAsymphony^®^ DSP Virus/Pathogen Midi Kit using a QIAsymphony robot (Qiagen), following the manufacturer’s instructions. Extraction was performed in duplicates; cfDNA was purified from two aliquots of fluid. The final elution volume was 30 µL per aliquot.

### 2.4 PNA‐Q‐PCR assay for mutation testing

In order to detect *EGFR* (exons 19, 20, 21)*, KRAS* (exon 2), and *BRAF* (exon 15) mutations (Table S1, list of mutations), we used a quantitative real‐time PCR (Taqman^®^) assay in the presence of a PNA clamp (Eurogentec, Seraing, Belgium) designed to inhibit the amplification of the wild‐type alleles (Gonzalez‐Cao *et al.*, 2018; Karachaliou *et al.*, 2015; Mayo‐de‐Las‐Casas *et al.*, 2017). The assay also allows estimation of the absolute and relative abundances of mutant alleles in positive samples. Briefly, amplification is performed in a final volume of 12.5 µL, using 3 µL (~ 4.5 ng) for exon 21 analysis or 1 µL (~ 1.5 ng) for exon 19 and p.T790M analysis of cfDNA; 6.25 µL of Genotyping Master Mix (Applied Biosystems, Foster City, CA, USA); 0.96 pmol of each primer; 1.2 pmol of probes and 6.25 pmol (for exon 21 and p. T790M) or 62.4 pmol (for exon 19) of PNA. Samples are submitted to 50 cycles of 15 s at 92 °C and 1.5 min at 60 °C, in a QuantStudio^TM^ 6 real‐time PCR System (Applied Biosystems/Thermo Fisher Scientific, Waltham, MA, USA). The sequence of the primers, probes, and PNAs used has been described elsewhere (Gonzalez‐Cao *et al.*, 2015; Karachaliou *et al.*, 2015). Analyses were carried out in duplicates using two samples of purified cfDNA, when possible. In addition, all samples were assayed in the absence of PNA to confirm the presence of cfDNA. Genomic DNAs from cell lines at 5 ng·µL^−1^ were used as positive and negative controls (Table S2). Extraction and nontemplate controls were added in each run. A sample was considered positive if the same mutant allele amplified in the two duplicates in the presence of PNA. If amplification was only detected in one duplicate, samples were reanalyzed and considered positive if again at least one of the duplicates was positive for the same mutated allele.

### 2.5 NGS for mutation testing

In order to detect the *ALK* resistance mutations and the G719X in *EGFR*, not covered by our Taqman assay (Table S3), we used next‐generation sequencing (NGS) with the GeneReader Platform^®^ (QIAGEN). Purified DNA (16.5 µL, ~ 40 ng) was used as a template to generate libraries for sequencing with the GeneRead^TM^ QIAact Lung DNA UMI Panel, according to manufacturer’s instructions. The panel is designed to enrich specific target regions containing 550 variant positions in 19 selected genes frequently altered in lung cancer tumors (*AKT1, ALK, BRAF, DDR2, EGFR, ERBB2/HER2, ESR1, KIT, KRAS, MAP2K1* *, MET, NRAS, NTRK1, PDGFRA, PIK3CA, PTEN, ROS1, FGFR1,* and *RICTOR*), including *MET* exon 14 skipping mutations. Libraries were quantified using a QIAxcel^®^ Advanced System, diluted to 100 pg·µL^−1^, and pooled in batches of 6 (liquid biopsies) or 12 (tissues). Clonal amplification was performed on 625 pg of pooled libraries by the GeneRead Clonal Amp Q Kit using the GeneRead QIAcube and an automated protocol. Following bead enrichment, pooled libraries were sequenced using the GeneRead UMI Advanced Sequencing Q kit in a GeneReader instrument. QIAGEN Clinical Insight Analyze software was employed to perform the secondary analysis of FASTQ reads, align the read data to the hg19 reference genome sequence, call sequence variants, and generate a report for visualization of the sequencing results. Variants were imported into the QIAGEN Clinical Insight Interpret web interface for data interpretation and generation of final custom report.

### 2.6 Quantification of the wild‐type and mutant alleles

For absolute quantification, serially diluted genomic DNAs from the appropriate positive or negative cell lines were analyzed by PNA‐Q‐PCR and the corresponding plot of log (genome equivalents per mL) vs. C*_t_* was represented. The resulting linear equation was used to interpolate the absolute concentration of mutant or wild‐type genomes in the samples based on the C*_t_* experimentally obtained (Fig. S1A). To determine the relative amount of mutations (allelic fraction), serial dilutions of positive in negative genomic DNA, corresponding to allelic fractions 1 : 20 to 1 : 20 000, were periodically run. In the PNA‐Q‐PCR assay, the difference between the C*_t_* of the mutated allele and the C*_t_* (*∆*C*_t_*) corresponds to a lower *EGFR* mutated allelic fraction, *∆*C*_t_ = a − *(*b* *× *log ML), where *ML* stands for the relative abundance of the mutant allele, or mutation load (Gonzalez‐Cao *et al.*, 2015; Mayo‐de‐Las‐Casas *et al.*, 2017). In consequence, we plotted the *∆*C*_t_* vs. the logarithm of the allelic fraction in the serial dilutions and used it to calculate the parameters *a* and *b* in the linear equation *∆*C*_t_ = a − *(*b × *log ML). Finally, the allelic fraction in the cfDNA samples was interpolated from this linear equation (Fig. S1B).

In the case of the NGS samples, the allelic fraction was directly estimated from the absolute amount of reads of the wt and mutant alleles.

### 2.7 Statistical analyses

The results were statistically analyzed using r software (R Core Team, 2012). Shapiro–Wilk normality tests revealed that the distributions of absolute and relative quantities of mutations in the fluid samples analyzed were not normal. In consequence, Kruskal–Wallis tests were used for comparisons and *P*‐values of < 0.05 were considered to be statistically significant.

## 3 Results

### 3.1 Patients and samples

**Figure 1:**
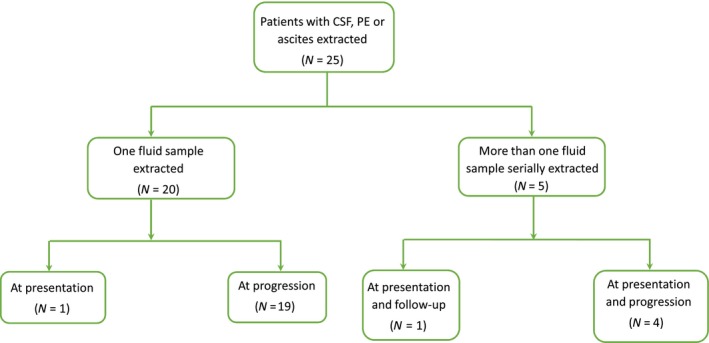
Flowchart of the patient population included in the study. None of the 25 patients had biopsies available at the time of fluid sample extraction.

A total of 42 fluid samples were collected from the 25 patients included in the study (Table 1, Fig. 1); eight ASC, 12 PE, and 22 CSF (Table 2). The type of fluid collected was dependent on the metastatic sites within the patient, cytologies were positive only in 10 cases. Of the 42 fluids included in the study, 23 had paired blood samples (four PE, 12 CSF, and seven ASC). Importantly, tissue biopsies at the moment of the fluid sample collection were not available in any case.

**Table 2:** Characteristics of the fluid samples included in the study.

Characteristics	All samples *N* = 42
Type of fluid	
Pleural effusion	12
Cerebrospinal fluid	22
Ascites	8
Cytology (presence of tumor cells)	
Positive	10
Negative	28
Not analyzed	4
Paired blood samples	
No	19
Yes	23
Pleural effusion	4
Cerebrospinal fluid	12
Ascites	7
Collection time and type of mutation	
Presentation	2
p.L858R	1
p.G12V	1
Follow‐up	6
p.G12V	4
Melanoma, p.V600E	2
Progression to 1st‐ and 2nd‐generation EGFR TKIs	14
*EGFR*‐sensitizing mutation	7
*EGFR*‐sensitizing mutation + p.T790M	7
Progression to 3rd‐generation EGFR TKIs	7
*EGFR*‐sensitizing mutation	4
*EGFR*‐sensitizing mutation + p.T790M	1
*EGFR*‐sensitizing mutation + p.T790M + p.C797S	2
Progression to ALKi	2
p.F1174C	1
p.L1196M	1
Progression to immunotherapy, p.V600E	6
Progression to BRAF + MEKi, melanoma, p.V600E	2
Progression to chemotherapy	3

Regarding the time of collection, two fluid samples were obtained baseline, six during to follow‐up, and 34 at progression to different therapies. One baseline sample corresponded to the CSF of a NSCLC patient and was positive for the p.L858R in *EGFR*. The remaining baseline and four follow‐up samples were CSFs from a NSCLC patient, all of them showed the p.G12V mutation in *KRAS* mutation but significant changes in the allelic fraction were observed (see below). Finally, the two additional follow‐up fluids were *BRAF* p.V600E‐positive ASC from a melanoma patient (Table 2).

Of the 34 fluids at progression included in the study, 14 were *EGFR*‐mut CSFs or ASC collected from NSCLC patients relapsing to first or second‐line TKIs. The p.T790M resistance was detected in 50% of them. Seven *EGFR*‐mut fluids corresponded to patients in progression to 3rd‐generation EGFR TKIs, the p.T790M had disappeared in four, while two were positive for the p.T790M + p.C797S. Two additional CSFs were collected from *EML4‐ALK* patients in relapse to TKIs, both showed *ALK* resistance mutations. Six ASC and two CSFs corresponded to melanoma patients progressing to immunotherapy and BRAF + MEK inhibitors, respectively, and they were all positive for the p.V600E mutation in BRAF. Finally, 3 fluids corresponded to NSCLC patients progressing to chemotherapy, *KRAS* mutations were detected in two cases (Table 2).

### 3.2 Comparison of fluid and plasma samples

**Figure 2:**
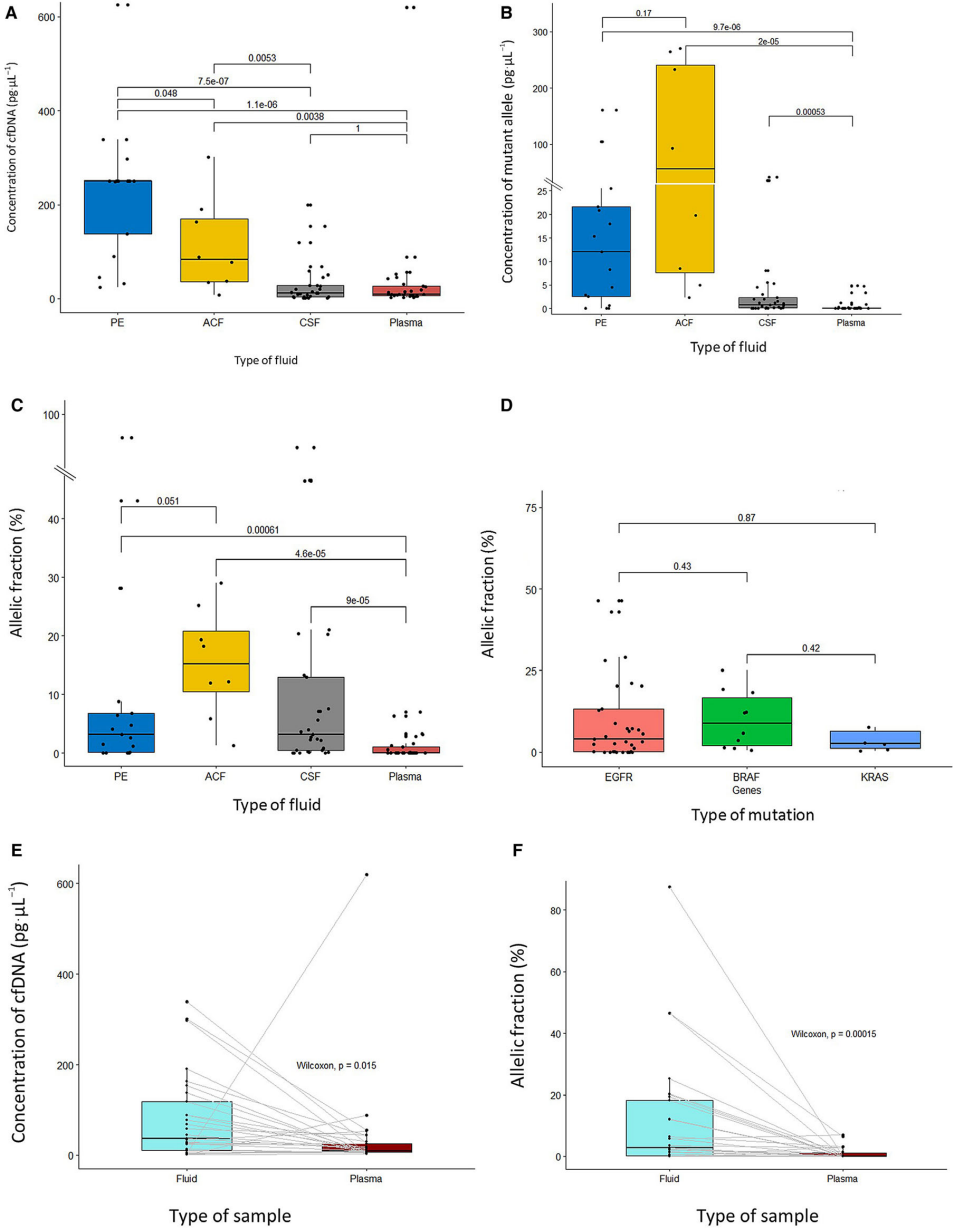
(A–C) Box plots representing (A) the dynamic ranges of total cfDNA, (B) the concentrations, and (C) the allelic fractions of mutant alleles in PE, CSF, ASC, and plasma samples. (D) Dynamic ranges of allelic fractions of *EGFR*, *BRAF,* and *KRAS* mutations in fluid samples. Solid lines indicate medians and numbers level of significance (*P*) in a Kruskal–Wallis test. (E, F) Box plot representing the dynamic ranges of (E) total cfDNA and (F) allelic fractions in fluid vs. plasma paired samples. Solid lines indicate medians and the numbers level of significance (*P*) in a paired Wilcoxon test.

The total concentration of cfDNA in the fluid (*n* = 42) and blood (*n* = 23) samples included in the study was estimated based on the C*_t_* of the wt alleles (see Materials and Methods). The concentrations of cfDNA in blood and CSF were found to be indistinguishable, with medians of 9.3 pg·µL^−1^ of plasma and 12.6 pg·µL^−1^ of CSF, respectively. In contrast, ASC and PE showed higher cfDNA concentrations, with medians of 82.1 and 250 pg·µL^−1^ of fluid, respectively. These differences were statistically significant in a Kruskal–Wallis test (Fig. 2A).

Absolute concentrations of the mutations were calculated from the C*_t_* of the mutant alleles and expressed as pg of mutant genomes per µL of fluid. We observed that ASC had the highest concentration of mutant genomes, with a median of 56.5 pg·µL^−1^; followed by PE, CSFs, and blood samples, with medians of 12.0, 0.7 and 0.002 pg·µL^−1^, respectively. The differences between plasma and the rest of fluids were significant in all cases (Fig. 2B).

The relative concentrations of mutations were determined based on the difference of C*_t_* between the wt and the mutant alleles. In contrast to absolute concentrations, we did not find statistically significant differences in the allelic fractions of the mutations in ASC, PE, and CSF, which showed medians of 15.2%, 3.2%, and 3.2%, respectively. However, the mutation loads in the three types of fluid were significantly higher than in plasma, where the median allelic fraction only reached 0.008% (Fig. 2C). When classifying samples according to the type of mutation, we did not observe significant differences in the allelic fractions of *KRAS*, *EGFR,* and *BRAF* mutations in the fluid samples (Fig. 2D).

### 3.3 Analysis of paired samples

Next, we specifically compared the results obtained in the 23 paired fluid and plasma samples. Of those, 14 were positive in both liquids and eight positive only in fluid. The remaining case was negative in the two liquids, while we did not find any case of positivity exclusively in plasma (Table S4). Interestingly, the eight cases negative in blood and positive in fluid corresponded to CSF samples extracted from patients with brain metastases.

The concentrations of cfDNA were usually higher in fluids vs. paired plasma samples, with medians of 37.2 and 9.5 pg·µL^−1^, respectively (Fig. 2E). Regarding the absolute and relative concentrations of mutant alleles, they were almost invariably superior in fluids vs. paired plasma; with medians escalating to 3.0 pg·µL^−1^ and 2.8% in fluids, compared to 0.002 pg·µL^−1^ and 0.008% in blood (Fig. 2F). These differences were significant in a paired Wilcoxon test.

### 3.4 Mutation analysis in fluids to guide treatment decisions

The results of the mutation analysis in fluids were used to guide clinical decisions (Table 3). In two *EGFR*‐mut patients progressing to first‐ or second‐generation EGFR TKIs with brain and pleural involvement, the p.T790M could be detected in CSF and PE, respectively. Both patients received osimertinib deriving a clinical benefit. Importantly, blood was negative for the resistance mutation in the patient with p.T790M‐positive CSF, who was in almost complete response to osimertinib for 18 months. In contrast, three *EGFR*‐mut patients progressing to first‐ or second‐generation EGFR TKIs only showed the sensitizing mutation in fluid samples. In consequence, chemotherapy or radiotherapy was selected, with partial responses or stabilization of the disease for 5–6 months. Paired blood samples were negative in most cases.

**Table 3:** Clinical decisions based on results of a mutation analysis in CSF, PE, or ascites. LF, loss to follow‐up; NA, paired blood not available for analysis; NEG, negative; NR, not recorded; VAF, variant allelic fraction; WBRT, Whole brain radiation therapy.

Patient code	Date of analysis	Type of fluid	Gen	Mutation status in fluid	VAF in fluid	Mutation status in blood	VAF in blood	Previous lines of treatment	Clinical decision	Type of response	PFS
6462	02/02/2017	CSF	EGFR	L858R+	5.6%	NEG	0%	1‐Erlotinib	Change to osimertinib	PR	18 months
				T790M+	2.3%		0%				
3970	23/11/2016	PE	EGFR	Del 24 pb+	0.2%	NA	–	1‐Erlotinib	Change to osimertinib	PR	9 months
				T790M+	0.2%						
4427	20/11/2017	CSF	EGFR	L858R+	46.4%	NEG	0%	1‐Gefitinib	Change to chemotherapy	SD	5 months
				T790M‐	0%		0%				
8415	27/05/2018	CSF	EGFR	E709K/G719S+ T790M‐	3.5% 0%	NA	–	1‐Afatinib	Change to chemotherapy	LF	LF
4827	22/09/2017	PE	EGFR	Del 15 bp+ T790M‐	2.7% 0%	Del 15 bp+ T790M‐	0.1% 0%	1‐Erlotinib	Lung radiotherapy and chemotherapy	PR	6 months
6606	26/10/2018	CSF	EGFR	Del 15 pb+ T790M‐ C797S‐	83.5% 0% 0%	NEG	0% 0% 0%	1‐Gefitinib 2‐Osimertinib	Change to chemotherapy	LF	LF
4249	03/05/2018	CSF	EGFR	Del 15 pb+ T790M‐ C797S‐	46.5% 0% 0%	NEG	0% 0% 0%	1‐Afatinib 2‐Osimertinib	Change to chemotherapy	SD	9 months
5057	20/06/2017	CSF	EGFR	Del 9 pb+ T790M‐ C797S‐	3.7% 0% 0%	Del 9 pb+ T790M‐ C797S‐	0.6% 0% 0%	1‐Erlotinib 2‐Olmutinib	Stop olmutinib Best supportive care	SD	6 months
6737^a^	10/03/2017	CSF	EGFR	Del 15 pb+ T790M+ C797S+ (cis)	16.7% 16.5% 11.2%	Del 15 pb+ T790M+ C797S+ (cis)	0.5% 0.3% 0.2%	1‐Erlotinib 2‐Osimertinib	Change to chemotherapy + avastin	SD	5 months
0965	25/09/2015	CSF	EGFR	L858R+ T790M‐ C797S‐	2.2% 0% 0%	NEG	0% 0% 0%	1‐Four lines of EGFR TKIs 2‐WBRT	Stop WBRT Change to best supportive care	SD	3 months
0145	17/04/2017	CSF	ALK	F1174C	4.0%	NEG	0%	1‐Seven lines of treatment 2‐Brigatinib	Change to lorlatinib	PR	22 months (ongoing)
8828	08/01/2019	CSF	ALK	L1196M	51.8%	NA	–	1‐Lorlatinib	Change to brigatinib	PR	4 months (ongoing)

a Patient presented in Fig. 3C.

Five of the CSF samples included in our study corresponded to patients progressing to 3rd‐generation TKIs, the sensitizing mutation was present but the p.T790M was either absent or present in *cis* configuration with the p.C797S. In consequence, osimertinib or olmutinib was discontinued and replaced by chemotherapy or best supportive care. In the case of chemotherapy, patients subsequently experienced stabilization of the disease for 5–9 months.

Two CSFs from patients in progression to brigatinib and lorlatinib were analyzed by NGS, showing *de novo* p.F1174C and p.L1196M mutations in ALK, respectively. New ALK TKIs were then selected based on their inhibitory profile against *ALK* resistance mutations. The patients have subsequently derived a significant clinical benefit, in both cases ongoing. Remarkably, a paired blood sample was negative in the case of the p.F1174C patient, who has been in response to lorlatinib for 22 months.

### 3.5 Mutation analysis of serial fluid samples

Two clinical cases where serial samples were obtained will described in further detail. The first case is a 63‐year‐old man, heavy smoker, diagnosed in February 2015 with a lung adenocarcinoma with leptomeningeal and medullary metastases and carrying a p.G12V mutation in *KRAS*. CSF samples were systematically collected and analyzed. The patient was referred to our hospital in November 2015; CSF was positive for the p.G12V mutation with a 2.8% allelic fraction while plasma was negative. Nab‐paclitaxel plus carboplatin and bevacizumab was administered with stabilization of the disease, which was accompanied by a reduction in the p.G12V mutation in CSF, which dropped to 0.5% in January 2016. However, a new CSF analysis on March 2016 showed a sharp increase to 7.6%. Imaging techniques subsequently revealed a radiological progression in brain. Stereotactic radiotherapy was then applied with a partial response and a new decrease of the mutation load to 0.8%. A second increase in the allelic fraction to 87.5% in July was accompanied with a rapid progression with leptomeningeal carcinomatosis and cauda equina syndrome. The patient was then administered immunotherapy in combination with radiotherapy, but was exitus in September (Fig. 3A).

**Figure 3:**
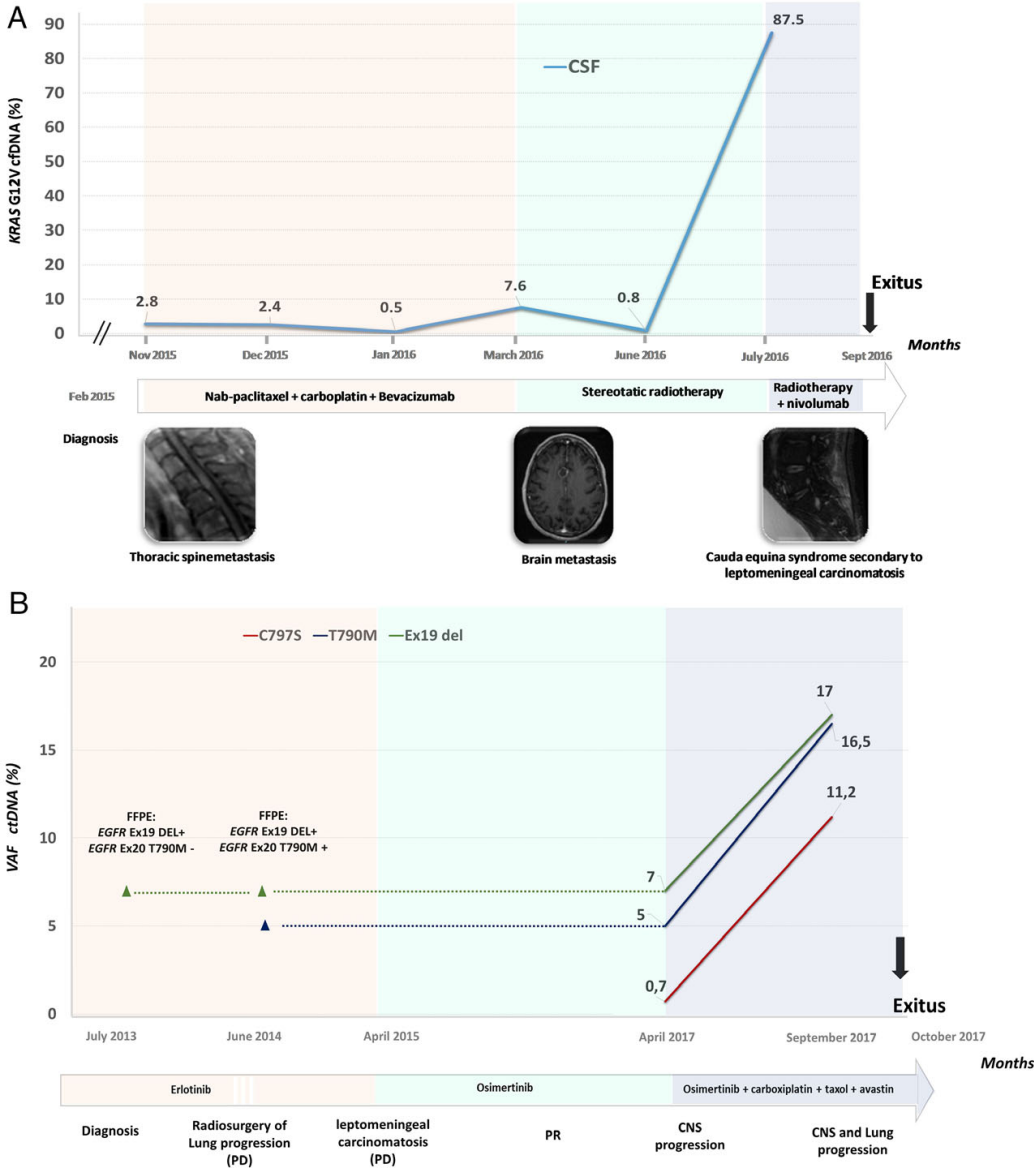
Mutation analysis in serial fluid samples from selected patients correlates with the course of the disease. (A) Evolution of the allelic fractions of *KRAS* p.G12V mutation in CSF of a 63‐year‐old man, heavy smoker, with a lung adenocarcinoma with leptomeningeal and medullary metastases. (B) Clinical course of a 62‐year‐old woman, never smoker, diagnosed in 2013 with stage IV NSCLC with bone and asymptomatic brain metastases at presentation.

The second case is a 62‐year‐old woman, never smoker, diagnosed in 2013 with stage IV NSCLC with bone and asymptomatic brain metastases at presentation, which harbored an *EGFR* deletion in exon 19 and received erlotinib. On June 2014, a single site of progression in lung was surgically resected; genetic analysis revealed the presence of the p.T790M. The patient remained with erlotinib until April 2015, when a significant impairment in performance status was accompanied by leptomeningeal carcinomatosis. Osimertinib was then administered and the patient remained in response for 2 years, when she presented a CNS progression. A first analysis of CSF, in April 2017, revealed the presence of the deletion in exon 19 and the p.T790M at allelic fractions of 7% and 5%, together with the p.C797S in cis with the p.T790M, at a 0.7% allelic fraction. This result indicated the emergence of a p.T790M + p.C797S tumor cell population resistant to osimertinib, which could not be rechallenged with other EGFR TKIs. Carboplatin, taxol, and avastin were then added to the treatment. A CSF sample collected 5 months later, in September 2017, presented the sensitizing mutation, the p.T790M and the p.C797S at significantly higher allelic fractions 12% and 16%, respectively. The patient underwent a rapid progression and was exitus a month later (Fig. 3B).

## 4 Discussion

Genetic testing in cfDNA purified from plasma of advanced cancer patients is quickly becoming a standard practice in many hospitals (Janku and Kurzrock, 2016; Janku *et al.*, 2017). However, PE, CSF, or ASC can also be employed for mutation testing. PE and ASC are usually available in large quantities, by minimally invasive procedures, in patients with pleural or peritoneal involvement. In contrast, CSF is more difficult to extract, and the volumes obtained are frequently limited. However, CSF is in direct contact with the brain and constitutes a valuable material for genetic testing in patients with SNC metastases or brain tumors (De Mattos‐Arruda *et al.*, 2015; Mouliere *et al.*, 2018; Nevel *et al.*, 2018; Panditharatna *et al.*, 2018; Pentsova *et al.*, 2016; Seoane *et al.*, 2019; Zhao *et al.*, 2016). Despite the potential of CSF, PE, or ASC as liquid biopsies, attention at the clinical level has been focused on blood and the performance and clinical utility of prospective molecular profiling in those fluids has not been adequately investigated.

In this study, we systematically collected and prospectively analyzed 22 CSF, 12 PE, and eight ASC samples obtained from advanced NSCLC and melanoma patients with pleural, peritoneal, and brain involvement. A majority of those ‘nonblood’ fluids were obtained at progression to different therapies, particularly TKIs. Importantly, patients could not be biopsied at the moment of sample collection, and fluids were the only material available. More than 50% of the CSF, PE, and ASC samples analyzed had negative cytologies and, in those where tumor cells were present, they were often insufficient for genetic analyses. Although some studies have reported more than 50% invalid samples when analyzing CSF from lung cancer patients (Kawahara *et al.*, 2019), the 42 fluid samples included in our study yielded valid results after centrifugation, cfDNA isolation, and mutation testing. Clinically relevant mutations could be detected in 41/42 cases (98%), a percentage significantly superior to the 70–80% sensitivity usually reported for mutation testing in cfDNA isolated from blood (Mayo‐de‐Las‐Casas *et al.*, 2017). In addition, mutation burden in fluid samples could be easily calculated, allowing for monitorization of the course of the disease in those patients where serial sampling was feasible. Our results are concordant with several retrospective studies, generally including low number of samples, reporting good sensitivity (64–100%) for *EGFR KRAS* or *BRAF* mutation detection in the CSF (Ballester *et al.*, 2018; Sasaki *et al.*, 2016; Yang *et al.*, 2014; Zhao *et al.*, 2016) or PE (Kang *et al.*, 2015; Liu *et al.*, 2014; Liu *et al.*, 2018; Shin *et al.*, 2017) of NSCLC and melanoma patients with brain or pleural involvement, either baseline or after progression to targeted therapies.

Of the 42 ‘nonblood’ liquid biopsies included in our study, 34 where obtained at relapse to antitumor therapies, constituting the largest reported cohort of fluid samples at progression. Clinically relevant mutations were detected in all the 34 samples, and the results of the testing were used to guide treatment decisions. A significant number of cases were CSFs or PEs from *EGFR*‐mut patients progressing to first‐ and second‐generation EGFR TKIs, where the presence of the p.T790M was used to select for osimertinib treatment. In contrast, when the EGFR‐sensitizing mutation was detected in significant allelic fractions but the p.T790M was absent, osimertinib was excluded. Although some studies suggest that the spatial heterogeneity in the distribution of the p.T790M can lead to positivity in the blood and negativity in the CSF of patients relapsing to EGFR TKIs (Hata *et al.*, 2014), in our cohort all cases p.T790M negative in CSF were also negative in blood. The fact that the majority of the patients with CSF analyzed were progressing exclusively in SNC might explain this finding. In addition to fluid samples at relapse to first‐ and second‐generation EGFR TKIs, CSF from seven patients with brain progression after treatment with third‐generation TKIs was prospectively tested. In two cases, only the initial sensitizing mutation was detected at allelic fractions as high as 45%, indicating disappearance of the p.T790M as a mechanism of resistance. In two other samples, the p.C797S was found in *cis* with the p.T790M, excluding rechallenge with EGFR TKIs (Chic *et al.*, 2017; Niederst *et al.*, 2015).

The results obtained in our paired samples confirm, in a prospective manner, that CSF, PE, and ASC are superior to blood for mutation testing in patients with SNC, pleural, and peritoneal involvement. The median allelic fractions of the mutations were 350 times higher in fluids than in paired blood samples and, in eight patients with SNC metastases, plasma samples tested negative but CSF was clearly positive for clinically relevant mutations. In addition, allelic fractions were below 0.01% in a significant number of positive paired blood samples, and mutations could only be detected because of the highly sensitive Q‐RT‐PCR used in our study. Commercially available kits and NGS panels would have probably missed some of them, due to insufficient sensitivity (Malapelle *et al.*, 2017; Mayo‐de‐Las‐Casas *et al.*, 2017). In contrast, the allelic fractions in nonblood fluids, with a median value of 2.8%, are adequate to be detected by these platforms. Our study also indicates that the results of mutation testing in fluids, not in blood, should be preferentially considered for the selection of antitumor treatments in cancer patients with particular metastatic sites. An *EGF*‐mut patient with brain metastasis negative for the p.T790M in blood but positive in CSF derived an 18‐month clinical benefit from osimertinib, while the detection of a p.F1174C mutation in an *EML4‐ALK* patient relapsing to brigatinib allowed for the selection of lorlatinib as the next‐line treatment (Gainor *et al.*, 2016) with a 22‐month response, still ongoing. Again, blood was negative for *ALK* mutations.

## 5 Conclusions

We have presented the results of the prospective mutation testing of 42 CSF, PE, and ASC samples from advanced NSCLC or melanoma patients with CNS, pleural, or peritoneal involvement. We have shown that fluids closer to metastatic sites are superior to blood for the detection of clinically relevant mutations and that genetic testing of these ‘nonblood’ fluids can be implemented in the routine clinical practice, being particularly useful in patients progressing to targeted therapies.

## Conflict of interest

The authors declare no conflicts of interest.

## Author contributions

SV, CMC, NJA, MGI, RR, and MAMV conceived and designed the study. CMC, NJA, MGI, BGP, RC, MJC, and MAMV conducted the experiments, acquired, and analyzed data. SVR, IMH, MGC, UM, AMB, NR, MM, RB, AB, XG, GT, NK, and RR provided clinical samples and collected clinical information. SV, CMC, and MAM wrote the manuscript. All authors reviewed and/or revised the manuscript.

## Supporting information


**Fig. S1.** Examples of linear plots used for absolute (A) and relative (B) quantification of mutant and wt alleles in liquid biopsy samples.
**Table S1.** Overview of reports on detection of mutations and other genetic alterations in cfDNA purified from fluids of advanced cancer patients.
**Table S2.** Cell lines used as positive controls in the PNA‐Q‐PCR assay. Negative controls were genomic DNAs from the BxPC‐3 or the PC‐3 cell lines.
**Table S3.** Mutations detected by the PNA‐Q‐PCR assay.
**Table S4.** Comparison of the mutational status in fluids and plasma in paired samples.
