# Supplementary material for: Prospective detection of mutations in cerebrospinal fluid, pleural effusion, and ascites of advanced cancer patients to guide treatment decisions
Source: Mol Oncol. 2019 Oct 11;13(12):2633–45. doi: 10.1002/1878-0261.12574 (PMC6887582; doi:10.1002/1878-0261.12574)
Supplement: Supplementary file 1 — Fig. S1. Examples of linear plots used for absolute (A) and relative (B) quantification of mutant and wt alleles in liquid biopsy samples. Table S1. Overview of reports on detection of mutations and other genetic alterations in cfDNA purified from fluids of advanced cancer patients. Table S2. Cell lines used as positive controls in the PNA‐Q‐PCR assay. Negative controls were genomic DNAs from the BxPC‐3 or the PC‐3 cell lines. Table S3. Mutations detected by the PNA‐Q‐PCR assay. Table S4. Comparison of the mutational status in fluids and plasma in paired samples. [file MOL2-13-2633-s001.docx]

**SUPPLEMENTARY FIGURES**

**Figure S1:** Examples of linear plots used for absolute (**A**) and relative (**B**) quantification of mutant and wt alleles in liquid biopsy samples

(A)

(B)

| **Technique** | **Samples**  **(n)** | **Primary tumor** | **Mutations analyzed** | **cfDNA from** | **mL of sample** | **% positive samples** | **LOD or Sensitivity** | **Ref** |
| --- | --- | --- | --- | --- | --- | --- | --- | --- |
| NGS (Nextseq500 sequencer) | 11 | Lung | Multiple | CSF | 10 mL | 81.8% | NR | (Zheng et al., 2019) |
|  |  |  |  | Plasma | 8 mL | 45.5% |  |  |
| NGS (Nextseq500 sequencer) | 23 | Lung | Multiple | CSF | 10 mL | 100% | NR | (Li et al., 2018) |
|  |  |  |  | Plasma | 8 mL | 73.1% |  |  |
| NGS (MSK-IMPACT platform) | 12 | Glioblas-toma | Multiple | CSF | 1-2 mL | 58%*  60.5%** | LOD: 2% | (De Mattos-Arruda et al., 2015) |
|  |  |  |  | Plasma | NR | 0%*  55.5%** |  |  |
| F-PHFA assay | 74 | Lung | *EGFR* | PE | NR | 44.4% | LOD: 1% | (Kawahara et al., 2018b) |
| cobas® EGFR Mutation Test v2.0 | 26 | Lung | *EGFR* | CSF | 1-2 mL | 33.3% Ex19  25% L858R  25% T790M | Sens: 87.5% | (Kawahara et al., 2018a) |
| ddPCR | 7 | Melanoma | *BRAF* | CSF | 0.75–1.8 mL | 57.1% | LOD: 0.1% | (Ballester et al., 2018) |
| NGS (ACHPv2) |  |  |  |  |  | 50% | LOD: 1% |  |
| Digital PCR (DigPCR) | 11 | Melanoma | *BRAF* | CSF | 1 mL | 55% | NR | (Momtaz et al., 2016) |
| Cycleave-time PCR assay | 7 | Lung | *EGFR* | CSF | NR | 100% | NR | (Sasaki et al., 2016) |
| ARMS-PCR assay | 30 | Lung | *EGFR* | CSF | 5 mL | 23% Ex19 del  20% L858R | Sens; 67% | (Yang et al., 2014) |
| HRM and Sanger sequencing | 36 | Lung | *EGFR* | PE | NR | 36.1% Ex19del  13.9% L858R, 2.8% Ex20 | LOD: 1-10% | (Lin et al., 2014) |

**SUPPLEMENTARY TABLES**

**Table S1:** Overview of reports on detection of mutations and other genetic alterations in cfDNA purified from fluids of advanced cancer patients. BL, broncoalveolar lavage; CSF, cerebrospinal fluid; ddPCR, droplet digital PCR; LOD, limit of detection; NGS, next generation sequencing; NR, not reported; PE, pleural effusion; Sens, sensitivity

*Including only patients with CNS disease

**Including all patients

**Table S2:** Cell lines used as positive controls in the PNA-Q-PCR assay. Negative controls were genomic DNAs from the BxPC-3 or the PC-3 cell lines

| Gen | Exon | Mutation | Positive cell line |
| --- | --- | --- | --- |
| *EGFR* | 19 | Del 15pb (p.E746-A750)  Del 9pb (p.L747-A750>P) | PC9  HCC4006 |
|  | 20 | p.T790M | NCI-H1975 |
|  | 21 | p.L858R | NCI-H1975 |
| *KRAS* | 2 | p.G12C  p.G13D | H23  DLD1 |
|  | 3 | p.Q61H | NCI-H460 |
| *BRAF* | 15 | p.V600E | HT29 |

**Table S3:** Mutations detected by the PNA-Q-PCR assay

| Gen | Exon | Mutations |
| --- | --- | --- |
| *EGFR* | 19 | p.L747-T751  p.L747-S753>S  p.E746-A750  p.L747-S752  p.E746-S752>V  p.E746-T751>A  p.L747-T751>P  p.L747-A750>P |
|  | 20 | p.T790M  p.C797S |
|  | 21 | p.L858R  p.L861Q |
| *KRAS* | 2 | p.G12C  p.G12A  p.G12V  p.G12S  p.G12D  p.G12R  p.G13C  p.G13D |
|  | 3 | p.Q61H  p.Q61L |
| *BRAF* | 15 | p.V600E  p.V600K |

**Table S4:** Comparison of the mutational status in fluids and plasma in paired samples

|  | **Plasma** | | |
| --- | --- | --- | --- |
| **Fluids** | **Mut +** | **Mut -** | **Total** |
| **Mut +** | 14 | 8 | 22 |
| **Mut -** | 0 | 1 | 1 |
| **Total** | 14 | 9 | 23 |

**SUPPLEMENTARY REFERENCES**

Ballester, L. Y., Glitza Oliva, I. C., Douse, D. Y., Chen, M. M., Lan, C., Haydu, L. E., Huse, J. T., Roy-Chowdhuri, S., Luthra, R., Wistuba, II, and Davies, M. A. (2018). Evaluating Circulating Tumor DNA From the Cerebrospinal Fluid of Patients With Melanoma and Leptomeningeal Disease. J Neuropathol Exp Neurol *77*, 628-635.

De Mattos-Arruda, L., Mayor, R., Ng, C. K., Weigelt, B., Martinez-Ricarte, F., Torrejon, D., Oliveira, M., Arias, A., Raventos, C., Tang, J.*, et al.* (2015). Cerebrospinal fluid-derived circulating tumour DNA better represents the genomic alterations of brain tumours than plasma. Nat Commun *6*, 8839.

Kawahara, A., Abe, H., Murata, K., Ishii, H., Azuma, K., Takase, Y., Hattori, S., Naito, Y., and Akiba, J. (2018a). Screening system for epidermal growth factor receptor mutation detection in cytology cell-free DNA of cerebrospinal fluid based on assured sample quality. Cytopathology.

Kawahara, A., Fukumitsu, C., Azuma, K., Taira, T., Abe, H., Takase, Y., Murata, K., Sadashima, E., Hattori, S., Naito, Y., and Akiba, J. (2018b). A Combined test using both cell sediment and supernatant cell-free DNA in pleural effusion shows increased sensitivity in detecting activating EGFR mutation in lung cancer patients. Cytopathology *29*, 150-155.

Li, Y. S., Jiang, B. Y., Yang, J. J., Zhang, X. C., Zhang, Z., Ye, J. Y., Zhong, W. Z., Tu, H. Y., Chen, H. J., Wang, Z.*, et al.* (2018). Unique genetic profiles from cerebrospinal fluid cell-free DNA in leptomeningeal metastases of EGFR-mutant non-small-cell lung cancer: a new medium of liquid biopsy. Ann Oncol *29*, 945-952.

Lin, J., Gu, Y., Du, R., Deng, M., Lu, Y., and Ding, Y. (2014). Detection of EGFR mutation in supernatant, cell pellets of pleural effusion and tumor tissues from non-small cell lung cancer patients by high resolution melting analysis and sequencing. Int J Clin Exp Pathol *7*, 8813-8822.

Momtaz, P., Pentsova, E., Abdel-Wahab, O., Diamond, E., Hyman, D., Merghoub, T., You, D., Gasmi, B., Viale, A., and Chapman, P. B. (2016). Quantification of tumor-derived cell free DNA(cfDNA) by digital PCR (DigPCR) in cerebrospinal fluid of patients with BRAFV600 mutated malignancies. Oncotarget *7*, 85430-85436.

Sasaki, S., Yoshioka, Y., Ko, R., Katsura, Y., Namba, Y., Shukuya, T., Kido, K., Iwakami, S., Tominaga, S., and Takahashi, K. (2016). Diagnostic significance of cerebrospinal fluid EGFR mutation analysis for leptomeningeal metastasis in non-small-cell lung cancer patients harboring an active EGFR mutation following gefitinib therapy failure. Respir Investig *54*, 14-19.

Yang, H., Cai, L., Zhang, Y., Tan, H., Deng, Q., Zhao, M., and Xu, X. (2014). Sensitive detection of EGFR mutations in cerebrospinal fluid from lung adenocarcinoma patients with brain metastases. J Mol Diagn *16*, 558-563.

Zheng, M. M., Li, Y. S., Jiang, B. Y., Tu, H. Y., Tang, W. F., Yang, J. J., Zhang, X. C., Ye, J. Y., Yan, H. H., Su, J.*, et al.* (2019). Brief Report: Clinical Utility of Cerebrospinal Fluid Cell Free-DNA as Liquid Biopsy for Leptomeningeal Metastases in ALK-Rearranged NSCLC. J Thorac Oncol.
